# A Novel Approach to Chemical Mixture Risk Assessment—Linking Data from Population‐Based Epidemiology and Experimental Animal Tests

**DOI:** 10.1111/risa.13323

**Published:** 2019-06-07

**Authors:** Carl‐Gustaf Bornehag, Efthymia Kitraki, Antonios Stamatakis, Emily Panagiotidou, Christina Rudén, Huan Shu, Christian Lindh, Joelle Ruegg, Chris Gennings

**Affiliations:** ^1^ Public Health Sciences Karlstad University Karlstad Sweden; ^2^ Icahn School of Medicine at Mount Sinai NY USA; ^3^ National and Kapodistrian University of Athens Athens Greece; ^4^ Stockholm University Stockholm Sweden; ^5^ Lund University Lund Sweden; ^6^ Karolinska Institutet Stockholm Sweden

**Keywords:** Chemical exposure, mixtures, risk assessment, sexual development

## Abstract

Humans are continuously exposed to chemicals with suspected or proven endocrine disrupting chemicals (EDCs). Risk management of EDCs presents a major unmet challenge because the available data for adverse health effects are generated by examining one compound at a time, whereas real‐life exposures are to mixtures of chemicals. In this work, we integrate epidemiological and experimental evidence toward a whole mixture strategy for risk assessment. To illustrate, we conduct the following four steps in a case study: (1) identification of single EDCs (“bad actors”)—measured in prenatal blood/urine in the SELMA study—that are associated with a shorter anogenital distance (AGD) in baby boys; (2) definition and construction of a “typical” mixture consisting of the “bad actors” identified in Step 1; (3) experimentally testing this mixture in an *in vivo* animal model to estimate a dose–response relationship and determine a point of departure (i.e., reference dose [RfD]) associated with an adverse health outcome; and (4) use a statistical measure of “sufficient similarity” to compare the experimental RfD (from Step 3) to the exposure measured in the human population and generate a “similar mixture risk indicator” (SMRI). The objective of this exercise is to generate a proof of concept for the systematic integration of epidemiological and experimental evidence with mixture risk assessment strategies. Using a whole mixture approach, we could find a higher rate of pregnant women under risk (13%) when comparing with the data from more traditional models of additivity (3%), or a compound‐by‐compound strategy (1.6%).

## 1 INTRODUCTION

Humans are exposed to suspected or proven endocrine disrupting chemicals (EDCs) that comprise a variety of chemical classes (e.g., phthalates, alkyl phenols, perfluorinated alkylated substances [PFASs]), with detectable concentrations from biomonitoring data in humans. An EDC has been defined as an exogenous chemical, or mixture of chemicals, that interferes with any aspect of hormone action with an adverse health outcome as a result (Zoeller et al., 2012). Accumulating evidence from human and animal studies indicates that early life exposure to EDCs may predispose the individual to disease at a later stage of life (Bergman, Heindel, Jobling, Kidd, & Zoeller, 2013). So far, health domains for which significant association with EDCs exposure has been shown in epidemiological studies include neurodevelopment, growth and metabolic outcomes, and sexual development (Bergman et al., 2013).

Epidemiology studies (generally observational studies evaluating single chemicals one at a time) demonstrate association of EDC concentrations with multiple adverse health outcomes. In contrast, experimental studies, typically *in vivo* animal studies, are used to establish a causative link between exposures to environmental chemicals and adverse effects. However, humans may be more or less sensitive to chemical exposures compared to animals, and experimental studies cannot reasonably represent all possible exposure patterns for humans. Therefore, it is the integration of observational human and experimental animal studies that may improve the scientific understanding of the implicated health effects as well as risk assessment of environmental chemicals.

The link between single chemical regulatory guideline values (e.g., points of departure [PODs] or reference doses [RfDs]) constructed from *in vivo* studies (EPA, 2000, 2007; Izadi, Grundy, & Bose, 2012) and concentrations in humans (based on biomonitoring) has been strengthened through the derivation and publication of biomonitoring equivalent (BE) values for dozens of important chemicals (Aylward, Kirman, Schoeny, Portier, & Hays, 2013). BE values are also called human biomonitoring values (HBM values) by the HBM Commission in Germany and the HBM4EU consortium in the European Union (Angerer, Aylward, Hays, Heinzow, & Wilhelm, 2011; Apel, Angerer, Wilhelm, & Kolossa‐Gehring, 2016). Establishment of a BE value is made in three steps. First, *in vivo* animal studies are used to estimate a dose–response relationship between a chemical and adverse effects recorded in experimental animals and a benchmark dose (BMD)—or a no‐observed‐adverse‐effect level (NOAEL)—is estimated. This gives the POD. Second, a “safe” dose is derived by dividing the POD from the experimental dose–response relationship with an uncertainty assessment factor (e.g., due to inter‐ and intraspecies differences, quality of the study, differences in duration of exposure). This gives the RfD. Third, toxicokinetic modeling is used to derive the BE value, i.e., the equivalent RfD in human biomonitoring data. The BE value may then be used for risk assessment since exposure to concentrations above the guideline values suggested by the BE is considered a risk in humans.

A margin of safety is calculated based on the distance between human exposure levels (e.g., median or 95th percentile of human concentrations) and these guideline values. Exposures close to the BE (and hence with a low margin of safety) or above the BE indicate a high risk of adverse health effects.

Since human exposure entails multiple EDCs in mixtures, the quantification of health effects associated with environmental mixtures is a critical goal for establishing environmental policy sufficiently protective of human health (Birgersson et al., 2017; Braun et al., 2017). A growing body of scientific data shows additive effects at lower doses than experimental effect thresholds for single compounds as described by the U.N. Environment Programme and World Health Organization (UNEP/WHO) (Bergman et al., 2013). This is also expressed in the concept of “something from nothing” (Silva, Rajapakse, & Kortenkamp, 2002). But even when single compounds have an effect alone, the assumption of dose addition necessarily indicates that a lower dose/concentration of each component combined is associated with the same effect, (e.g., Gennings et al., 2005). Hence, health risks linked to EDC exposure may be significantly underestimated because research and risk evaluations conducted in this field almost exclusively have used a single‐compound approach.

Two strategies for linking human exposures to mixtures and the regulatory guideline values include (1) a whole mixture strategy based on an experimentally tested reference mixture and mixtures sufficiently similar to the reference mixture (EPA, 2007) and (2) consideration of a hazard index (HI) where the components of a mixture are assumed to contribute additively to the observed toxicity (i.e., following the assumption of dose addition) (EPA, 2007; Lioy et al., 2015).

The impact of EDCs on male reproductive health has been a research focus for more than 20 years. During this period, multiple studies on laboratory animals (Sharpe et al., 1995) and humans (Swan et al., 2005) have demonstrated the sensitivity of the developing male reproductive system to several phthalates. Phthalates can reduce the production of androgens by the testis and rodent studies have demonstrated that diethyl hexyl phthalate (DEHP) and dibutyl phthalate (DBP) disrupt androgen signaling when administered in the critical window for the development of the reproductive tract (Macleod et al., 2010; Van den Driesche et al., 2011). There is also experimental evidence that mixtures of EDCs combine additively in rodents (Hass et al., 2007; Howdeshell et al., 2015; Lioy et al., 2015; Metzdorff et al., 2007; Rider et al., 2009). In humans, phthalates (e.g., DEHP and DBP) are suspected to cause male reproductive and developmental abnormalities (ECHA, 2013).

Anogenital distance (AGD)—the distance from the anus to the genitals—is a marker that is used in animal studies to assess reproductive toxicity (EPA, 1996). AGD is a sexually dimorphic trait that develops *in utero* under androgen control. AGD is 50–100% longer in males than females in both humans and animals (Salazar‐Martinez, Romano‐Riquer, Yanez‐Marquez, Longnecker, & Hernandez‐Avila, 2004).

Numerous studies have shown that prenatal phthalate exposure (notably DEHP, DBP, and butyl benzyl phthalate [BBzP]) shortens the AGD in male rodents (Foster, 2005; Van den Driesche et al., 2011). Only a few human studies have examined prenatal phthalate exposure and AGD. The first to examine this association in humans reported a significant inverse relationship between male AGD and prenatal exposure to diethyl phthalate (DEP), DBP, BBzP, and diisobutyl phthalate (DiBP) metabolites (Swan et al., 2005), i.e., the higher the exposure to these phthalates, the shorter the AGD in the male babies. A later publication with a larger sample size and more powerful statistical methods also found an association between male endpoints and metabolites of DEHP (Swan, 2008). A relationship between prenatal DEHP exposure and shorter AGD in male newborns has also been reported from Japan (Suzuki et al., 2012) and from Mexico (Bustamante‐Montes et al., 2013). Recently, a Swedish study found a significant association between prenatal exposure to di‐isononyl phthalate (DiNP) and shorter AGD in boys at 21 months of age (DiNP has been replacing DEHP as a plasticizer in soft PVC) (Bornehag et al., 2015). In contrast, a Danish study with lower phthalate concentrations than in the Swedish study did not find an association between prenatal phthalate exposure and AGD in children (Jensen et al., 2015).

Thus, there are limited data indicating that prenatal exposure to phthalates may reduce AGD in male children. However, there is no human study available focusing on prenatal exposure to mixtures of EDCs and AGD in children. There is also a lack of data based on an integrated approach including both population‐based epidemiology and experimental animal tests for risk assessment of mixture exposures.

### 1.1 Study Objective

The current study aims to set the stage for a novel approach to risk assessment of chemical mixtures related to endocrine disruption, using as a paradigm their effects on male sexual development in both young boys and male mice. For this purpose, this case study will identify a reference mixture that is associated with AGD changes in a population of boys from a pregnancy cohort, then identify the dose–response relationship and POD for this reference mixture in mice and the related BMD in humans (BE), and finally integrate human and mice data for risk evaluation using a whole mixture and a dose‐addition approach.

## 2 METHODS

The overall concept of the study is to use a four‐step procedure. In a first step, we identify chemicals in real‐life mixtures of EDCs, measured prenatally in a human pregnancy cohort, the Swedish Environmental Longitudinal, Mother and Child, Asthma and allergy (SELMA) study, that are associated with AGD reduction in baby boys. We call these chemicals the “bad actors.” In a second step, we define and construct a “typical” mixture of these “bad actors” by mixing them according to the geometric mean concentrations established in the measured mixtures. In a third step, we experimentally test this “typical” mixture in an *in vivo* mouse model in order to identify the dose–response relationship for the mixture and define a BMD to use as a POD in the risk evaluation. Finally, in a fourth step, we test for sufficient similarity (Marshall et al., 2013; with a more general discussion in Catlin et al., 2018) between the experimentally observed reference mixture and those from biomonitoring data per study subject. For those determined to be sufficiently similar, we compare the POD from the animal data to human exposure data to derive a risk quota and ultimately a risk index, the “similar mixture risk indicator” (SMRI).

Hence, in this proposed approach, we rely on a whole mixture approach based on the concept of sufficient similarity, which we term a “Similar Mixture ApproaCH” (SMACH). We compare the results from SMACH to the component‐based approach under the assumption of dose addition, i.e., comparison of the SMRI of sufficiently similar mixtures to the HI under the assumption of additivity, described in more detail below.

### 2.1 Step 1: Identification of “Bad Actors” in Human Data

The aim of Step 1 was to identify prenatal EDCs (i.e., “bad actors”) associated with AGD in boys using epidemiological data in a pregnancy cohort.

The SELMA study is a pregnancy cohort study designed to investigate early life exposure to environmental chemicals and health outcomes related to growth, developmental, and chronic diseases for the children (Bornehag et al., 2012). SELMA recruited pregnant women in the county of Värmland, Sweden, between September 2007 and March 2010. Women who could read Swedish and were not planning to move out of the county were recruited at their first antenatal care visit: 8,394 pregnant women were identified, 6,658 were eligible, and 2,582 (39%) agreed to participate. Detailed recruitment selection criteria and sample collection procedures have been published previously (Bornehag et al., 2012).

The decision on selection of compounds analyzed in prenatal urine and serum in the SELMA study was taken long before these current analyses were conducted. In a first set of compounds, we included phthalates, alkyl phenols, and perfluorinated compounds because these compounds have been shown to have effects on experimental animal models and to be associated with health and development in human data.

For the current study, we have prenatal creatinine‐adjusted phthalate metabolite and bisphenol A (BPA) and triclosan levels measured in urine and eight PFASs in serum (in total 20 metabolites or compounds), AGD measurement in 184 boys when they were 21 months old, data on covariates for the biostatistical modeling, and 2,313 pregnant women in the complete case analyses of exposures.

#### 2.1.1 Analyses of Chemicals in Prenatal Urine and Serum

A first morning void urine sample and serum were obtained from the 184 pregnant women in weeks 3–27 of pregnancy (median week 10, and 96% of the samples were taken before week 13) at enrollment in the study (Bornehag et al., 2015).

Urine samples were collected in supplied glass containers at home and transferred into polypropylene tubes, without any other assisting equipment, for easy transportation. Samples were stored at –20 °C before being processed in the laboratory at the Division of Occupational and Environmental Medicine, Lund University, Sweden. Quantitative analysis was performed for urinary phthalates (10 metabolites from five parent compounds, bisphenol A, and triclosan; Table II). Urinary concentrations were quantified using a triple quadrupole linear ion trap mass spectrometer (QTRAP 5500; AB Sciex, Foster City, CA, USA) coupled with a liquid chromatography system (UFLCXR, Shimadzu Corporation, Kyoto, Japan; LC/MS/MS). The samples were prepared and analyzed according to the method presented in Bornehag et al. (2015) and Gyllenhammar et al. (2017). Urinary creatinine concentrations were analyzed according to an enzymatic method described by Mazzachi, Peake, and Ehrhardt (2000).

The analyses of perfluoroheptanoic acid (PFHpA), perfluorohexane sulfonate (PFHxS), perfluorooctane sulfonate (PFOS), perfluorooctanoic acid (PFOA), perfluorononanoic acid (PFNA), perfluorodecanoic acid (PFDA), perfluoroundecanoic acid (PFUnDA), and perfluorododecanoic acid (PFDoDA) were performed using liquid chromatography–tandem mass spectrometry (LC/MS/MS) at the Department of Occupational and Environmental Medicine in Lund, Sweden. A detailed description of the method is presented in Lindh et al. (2012). Briefly, aliquots of 100 µL serum were added to 25 µL of a water:acetonitrile (50:50) solution containing labeled internal standards. Proteins were precipitated by acetonitrile and vigorously shaking for 30 minutes. The samples were then centrifuged and the supernatant was analyzed using a LC (UFLCXR, SHIMADZU Corporation, Kyoto, Japan) connected to a hybrid triple quadrupole linear ion trap mass spectrometer (QTRAP 5500, AB Sciex). The analyses of PFOA and PFOS are part of the Round Robin Intercomparison Program (Professor Dr. Med. Hans Drexler, Institute and Out‐patient Clinic for Occupational‐, Social‐ and Environmental Medicine, University of Erlangen‐Nuremberg, Germany) and the results were within the tolerance limits.

#### 2.1.2 Measurements of AGD in Boys

Measurements of AGD were made in 225 baby boys at 21 months of age (Bornehag et al., 2015), of which 184 had all data for the current analyses. Two AGD measures were used, a longer AGD measurement (AGDap) is measured from the center of the anus to the anterior base of the penis, and a shorter (AGDas) from the center of the anus to the posterior bases of the scrotum. Each AGD was measured three times and the average value (mm) is reported. In this work, we focus on AGDas because no association was found with AGDap in the single‐compound analyses conducted earlier (Bornehag et al., 2015).

#### 2.1.3 Statistical Analysis of Human Data

Our primary analysis in Step 1 was weighted quantile sum (WQS) regression (Carrico, Gennings, Wheeler, & Factor‐Litvak, 2015), which focuses the inference in a single direction, i.e., a shorter AGDas with increased exposure to mixtures of the 20 compounds). We analyzed three different mixtures separately: urinary compounds (*N* = 12), serum compounds (*N* = 8), and all other compounds (*N* = 20).

In short, WQS regression is a strategy for estimating empirical weights for a weighted sum of quantiled concentrations (e.g., quartile or decile scores) most associated with the health outcome, i.e., ∑_*j*_ *w_j_q_j_*, where *w_j_* is the estimated weight for the *j*th compound and *q_j_* is the quantile score for the given subject (e.g., for quartile scores, *q* = 0, 1, 2, or 3). The results are a beta coefficient associated with the weighted sum (estimate, *SE*, and *p* value) and the empirical weights (which are constrained to sum to 1). The components most associated with the health outcomes have nonnegligible weights. Components from a set of *c* components with weights above 1/*c* were designated as “bad actors.” For example, with 12 phthalates and phenols, we use a threshold of interest of 1/12 = 0.08.

Preliminary analyses of potential covariates/confounders included evaluation of the potential association between questionnaire variables and biomonitoring concentrations. In addition, variables were included in models based on evidence in the literature.

The SELMA study was approved by the Ethical Board of Uppsala, Sweden.

### 2.2 Step 2: Definition and Construction of a Reference Mixture of “Bad Actors”

The aim of Step 2 was to construct a typical mixture consisting of “bad actors” defined in Step 1 using human biomonitoring data in SELMA.

Following Koch et al. (2007), we calculated the daily intake (DI) of the identified “bad actors” from Step 1 using urinary geometric mean concentrations in 2,313 SELMA mothers for estimating the mixing proportions of “bad actors,” i.e., a reference mixture called Mixture S. The model for DI includes the creatinine‐related metabolite concentrations together with reference values for the creatinine excretion (David, 2000) as follows:

(1) DI[ mol /kg bw / day ]=concmetabolite[g/L]MWmetabolite[g/ mol ]Creatinine[g crt /L]×CE[g crt /kg bw / day ]FUE=UE[ mol /g crt ]×CE[g crt /kg bw / day ]FUE,

where

- Creatinine[g/L]=Creatinine[ mmol L]×113.12[g mol ]×[ mol 1,000 mmol ];
- UE is the molar urinary excretion of the respective metabolite(s); and
- CE is the creatinine excretion rate normalized by body weight (BW). Following the CHAP report (Lioy et al., 2015), we set CE to 0.023 g/kg/day for these pregnant women.
- The molar fraction *F*_UE_ describes the molar ratio between the amount of metabolite(s) excreted in urine and the amount of parent compound taken up.

We estimated the serum levels of the phthalates from the estimated DI of the diesters to construct mixing proportions, following a simplified equation of a one‐compartment toxicokinetic model assuming a total bioavailability and resorption from the intestine (Fromme et al., 2007):

(2) E( mol /kg bw )=0.693t1/2×VdLkg BW ×Cp mol L,

where *t*_1/2_ is the half‐life (set at 0.4 days), *V_d_* is the volume of distribution (set at 0.2 L/kg_BW_), and *C_p_* is the blood plasma concentration resulting from a given exposure dose (*E*). Solving for *C_p_* in molar units,

(3) Cp( mol /L)=E[ mol /kg bw ]×t1/20.693×1Vd[L/kg bw ].

### 2.3 Step 3: Experimental Animal Tests of the Reference Mixture

The aim of Step 3 is to determine a dose–response relationship in male mice exposed prenatally to Mixture S, and to evaluate a BMD.

The *in vivo* study was conducted in C57/BL6 mice. The breeders were purchased from the Hellenic Pasteur Institute (Athens, Greece) and left to acclimate in the animal vivarium (Biology‐Biochemistry Lab, Faculty of Nursing, National and Kapodistrian University of Athens) before mating. Animals were kept under standard housing conditions (12‐hour light/dark cycle, 50 ± 5% relative humidity, 22 ± 2 °C) in polypropylene cages and were offered phytoestrogen‐deficient diet (Altromin1324P, Germany) and tap water *ad libitum*. Pregnant mice were exposed daily, throughout gestation, to Mixture S, comprising the chemicals identified as “bad actors” from Step 1 and in mixing proportions as determined in Step 2. The pregnant dams were treated with three doses of Mixture S (0.5*X*, 10*X*, 100*X*) or the vehicle (DMSO in PBS), where *X* refers to the estimated geometric mean of SELMA mothers' levels for the chemicals in Mixture S. The daily dose was offered via an organic corn flake to individually caged females and was adjusted to their BW gain. DMSO exposure did not exceed 0.25 µL/g BW. At weaning (postnatal day 21, PND21), the AGD and BW were recorded in mice by one and the same observer, who was unaware of the treatment group. AGD was measured using a digital caliper as the length of the perineum from the center of the genital papilla to the center of the anus (Manno III, 2008).

All animal handling and experimentations were done in accordance with the European Communities Council Directive of September 22, 2010, on the protection of animals used for scientific purposes (2010/63/EU) and the experimental protocol was approved by the Ethical Committee of the Prefecture of Attica‐Veterinary Department (approval number 4783). All efforts were made to minimize animal suffering and to reduce the number of animals used.

#### 2.3.1 Statistical Analyses of Animal Data

Offspring of at least 4 L per dose were used in the analysis with litters identified by dam IDs. A mixed effects (with random effects associated with litters) ANOVA was initially used to determine treatment effects of the various Mixture S doses on the mean AGD/BW in comparison to the control group mean, using Dunnett's test to adjust for multiple testing. Subsequently, a quadratic mixed effects dose–response model was fit to the AGD/BW data, parameterized based on the results from the Dunnett's test, where *x* represents the log10(concentration +1) of the total dose of Mixture S, i.e.,

(4) $$\mu =\beta_{0}+\beta_{1}x+\beta_{2}x^{2}.$$

Intraliter observations were assumed to be correlated with interliter observations assumed independent.

The benchmark response (BMR) was set as a 5% decline and the BMD (Filipsson et al., 2003) was found using the quadratic formula. The variance of the estimated BMD was calculated using the delta method in PROC NLMIXED in SAS.

We assumed that an additional 3% decline from the BMR of 5% would be considered a similar effect size—thereby defining a change in the total dose of Mixture S (an effective dose of 8% decline minus the BMD associated with a BMR of 5%) considered to be sufficiently similar.

### 2.4 Step 4: Risk Evaluation Using a Whole Mixture Approach

The aim of Step 4 is to compare the BMD from the animal data to human exposure data for risk evaluation. Hence, in this step, we rely on a whole mixture approach based on the concept of sufficient similarity and a dose‐addition approach.

#### 2.4.1 Sufficient Similarity Approach

Using the “poor data case” from Marshall et al. (2013), the estimated distance between the BMD of each SELMA pregnant woman's mixture and the reference mixture (Mixture S) was calculated as:

(5) $${\hat{d}}_{i}={\hat{T}}_{r}\sqrt{\sum_{j=1}^{4}{\left(a_{ij}-a_{rj}\right)}^{2}},$$

where *T_r_* is the BMD of the reference mixture in terms of total dose, with the *i*th subject's mixture and the reference mixture proportions given by the *a_ij_* and *a_rj_*, for the *j*th component, respectively. The upper one‐sided confidence limit (UL) on the distance is given by:

(6) d^i+t0.95,df=N−pSE(d^i), where SE(d^i)=SE(T^r)∑j=14aij−arj2.

The radius (*R*) of the similarity region for the test for sufficient similarity was based on the difference between the estimated BMD and the ED associated with a 8% decline. Following Marshall et al. (2013), when UL < *R*, the BMD based on the mixture as measured from the *i*th subject is claimed to be sufficiently similar to the reference mixture BMD. The proportion of pregnant women with mixtures claimed to be sufficiently similar to the reference mixture has been reported. For these subjects, the SMRI was calculated using their estimated phthalate diesters (*E_j_*, *j* = 1,…, 4) and the reference mixture proportions (*mRV_j_* = *a_rj_*BMD), where SMRI values exceeding 1 are of concern:

(7) $${\mathrm{SMRI}}_{i}=\sum_{j=1}^{4}\frac{E_{j}}{mRV_{j}}.$$

#### 2.4.2 Dose‐Addition Approach

A standard comparison strategy for cumulative risk assessment of co‐occurring chemicals is to group them into sets that combine additively. There is evidence in the literature that the effect of phthalates on AGD approximates a dose‐addition model (Lioy et al., 2015). In that light, we constructed an HI per subject based on the measured concentrations of the four phthalates in Mixture S and published RfDs, i.e., BE values (Aylward et al., 2013).

## 3 RESULTS

### 3.1 Step 1: Identification of “Bad Actors” in Human Data

We used WQS regression to evaluate the mixture effect of the 20 compounds on AGDas in 184 boys, adjusted for the covariates as described in Table I. The WQS index was not significant (95% CI = [−0.74, 1.90]; *p* = 0.390) when all 20 analytes were included. Neither was the index was significant (95% CI = [−0.74, 1.90]; *p* = 0.390) when only the eight serum‐based PFASs were included. However, the WQS index including phthalates and alkyl phenols, the empirically weighted sum of metabolite concentrations (Table II), was significantly associated with a decrease in AGDas (*p* = 0.015).

**Table I:** Summary Statistics for Dependent Variables and Covariates for 184 Boys in the SELMA Study

Dependent Variable	Mean (*SD*)
AGDas (mm)	41.4 (6.1)
Covariates	
Child age at AGD evaluation (months)	21 (1.6)
Child weight for age (percentile)	56 (27)
Gestational week for urine sampling	10 (2.3)
Mothers urinary creatinine (mmol/L) median (IQR)	9.8 (6.0)

**Table II:** Single Chemical (Log Concentration Scale) and WQS Regression Results for 184 Mother–Child Pairs for Analyses of AGDas Adjusted for Covariates as Described in Table I

			Single Chemical Analyses	WQS Analyses (Weights)
Compounds and Metabolites			Beta (*p*‐Value)	Beta: −1.6 (*SE* = 0.65) *p* = 0.015
Phthalates	DEP	MEP	0.61 (0.522)	0.04
	DBP	MBP	−1.4 (0.346)	0.09
	BBzP	MBzP	−1.6 (0.090)	0.29
	DEHP	MEHP	−1.3 (0.289)	0.08
		MEHHP	−1.2 (0.364)	0.01
		MEOHP	−0.77 (0.570)	<0.01
		MECPP	−0.89 (0.527)	0.01
	DINP	MHiNP	−1.6 (0.024)	0.30
		MOiNP	−1.8 (0.026)	0.13
		MCiOP	−1.5 (0.081)	0.04
Phenols	BPA		1.2 (0.307)	<0.01
	Triclosan		0.88 (0.146)	0.01

DEP, di‐ethyl phthalate; MEP, mono‐ethyl phthalate; DBP, di‐butyl phthalate; MBP, mono‐butyl phthalate; BBzP, butyl‐benzyl phthalate; MBzP, mono‐benzyl phthalate; DEHP, di‐ethyl‐hexyl phthalate; MEHP, mono‐ethyl‐hexyl phthalate; MEHHP, mono‐ethylhydroxy‐hexyl phthalate; MEOHP, mono‐ethyl‐oxo‐hexyl phthalate; MECPP, mono‐ethyl‐carboxy‐pentyl phthalate; DINP, di‐isononyl phthalate; MHiNP, mono‐hydroxy‐iso‐nonyl phthalate; MOiNP, mono‐oxo‐iso‐nonyl phthalate; MCiOP, mono‐carboxy‐iso‐octyl phthalate; BPA, bisphenol A.

The metabolites associated with the sum of DiNP and BBzP accounted for 47% and 29% of the weight, respectively, in the analysis of AGDas. In summary, the strongest signals associated with a decrease in AGDas for boys were from DBP, BBzP, DEHP, and DiNP metabolites (i.e., those compounds that were deemed as “bad actors”).

### 3.2 Step 2: Definition and Construction of a Reference Mixture of “Bad Actors”

In order to estimate the external doses of the “bad actors”—to be used for dosing in the mouse study—we used Equation (1) and estimated the DI of the phthalate diesters (using Table III) for each pregnant woman (*N* = 2,313) in the SELMA study. That is, four metabolites were used to estimate DEHP exposures, three metabolites were used to estimate DINP exposures, and single metabolites were used for DBP and BBzP (Table III). The geometric means of the estimated diesters in serum were used to derive proposed environmentally relevant mixing proportions for the reference mixture (Mixture S), as described in Table IV.

**Table III:** Molecular Weights and Excretion Factors Used for Calculating Diester Daily Intake in SELMA Women

Diester	Diester (MW)	Metabolite	Metabolite (MW)	Excretion Factor (*F*_UE_)
DEP	222	MEP	194	69%
DBP	278	MBP	222	69%
BBzP	312	MBzP	256	73%
DEHP	391	MEHP	278	45.2%
		MEHHP	294	
		MEOHP	292	
		MECPP	308	
DINP	419	MHiNP	308	29.8%
		MOiNP	306	
		MCiOP	322	
BPA	228			54%
Triclosan	290			54%

**Table IV:** Mixing Proportions of Phthalates for Mixture S Determined from the Geometric Means of Estimated Intakes in Serum from Identified “Bad Actors” in the Analysis of AGD

Phthalate (Metabolite)	Estimated Geometric Mean in SELMA (1*X*) (mol/L)	Mixing Proportions
DBP (MBP)	2.3 E‐08	0.33
BBzP (MBzP)	1.1 E‐08	0.16
DEHP (MEHP)	1.5 E‐08	0.21
DINP (MINP)	2.1 E‐08	0.30
SUM	7.0 E‐08	1.0

### 3.3 Step 3: Experimental Animal Tests of the Reference Mixture

**Figure 1:**
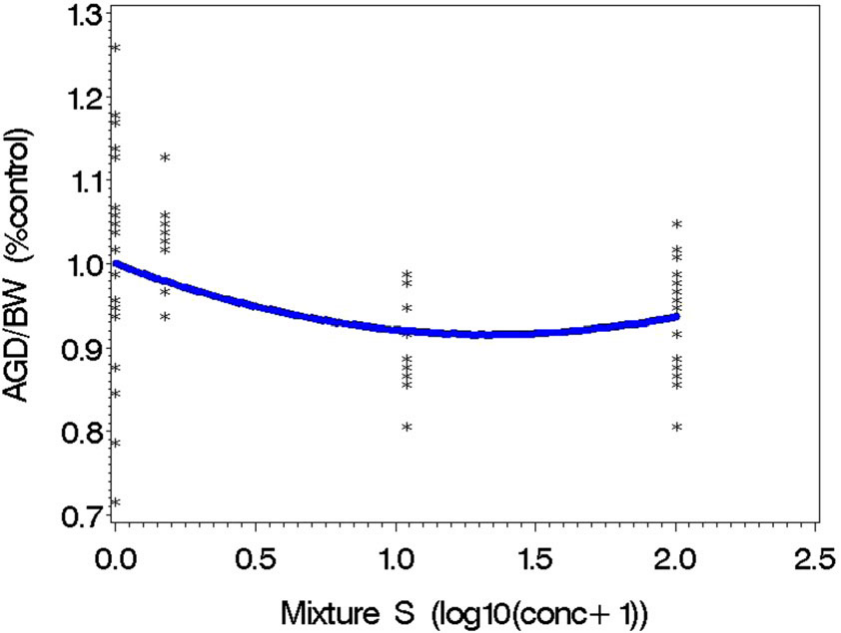
Observed and predicted dose–response relationship between Mixture S and AGD/BW in male mice. Significant differences for AGD/BW are shown in Table V.

The dose responsiveness was tested on male mice exposed prenatally to Mixture S. Summary statistics from the mouse study are provided in Table V. There was a significant difference among the four group means in a mixed effects ANOVA model (*p* < 0.001; data not shown), but only the 10*X* and 100*X* group means were significantly lower than the control mean (Fig. 1), adjusting for multiple testing, using Dunnett's test with a family‐wise 5% significance level.

**Table V:** Summary Statistics for AGD/BW from the Mice Study of Mixture S (Fig. 1) and Estimated EDs of AGD/BW on Log10(Conc + 1) Scale Where Concentration Is Relative to Typical Mixture in SELMA (i.e., 1*X*)

Dose for Mixture S	AGD/BW Mean (*SD*)	*N*
0X (DMSO) (Control)	0.99 (0.13)	24
0.5X	1.02 (0.05)	9
10X	0.89 (0.05)^a^	13
100X	0.94 (0.06)^a^	19
	Estimate (*SE*)	BMDL
BMD with BMR = 0.95	0.49 (0.33)	−0.09
ED (0.92)	1.05 (1.30)	
Similarity region radius	0.56	

a Significantly different (*p* < 0.05) from control using a Dunnett's test for multiple testing.

To estimate a BMD, a quadratic model was used to determine the dose–response relationship between AGD/BW (% control) and log10 (concentration +1) of the mixture (Fig. 1). With a BMR of a 5% decline in AGD/BW, the estimated BMD was 0.49 on the log scale (i.e., 10^0.49^ – 1 = 2.1*X* of “typical” SELMA exposure). The estimated effective dose (ED) associated with an 8% decline was 1.05 (i.e., 10.2*X*). We then determined the radius of the similarity region (on the log scale of 1*X* +1) to be *R* = 1.05 − 0.49 = 0.56.

### 3.4 Step 4: Risk Evaluation Using a Whole Mixture Approach

We first determined the proportion of SELMA pregnant women who have mixing proportions that were considered to be sufficiently similar to the reference mixture (Mixture S). We accomplished this by calculating the upper confidence limit on the distance between each human subject's estimated BMD in the data‐poor case, Marshall et al. (2013), and the estimated BMD from the mouse study of Mixture S (Table V). Our results indicate that 85% of the SELMA women (*N* = 1,958 out of 2,313 women) have mixtures sufficiently similar to Mixture S.

**Figure 2:**
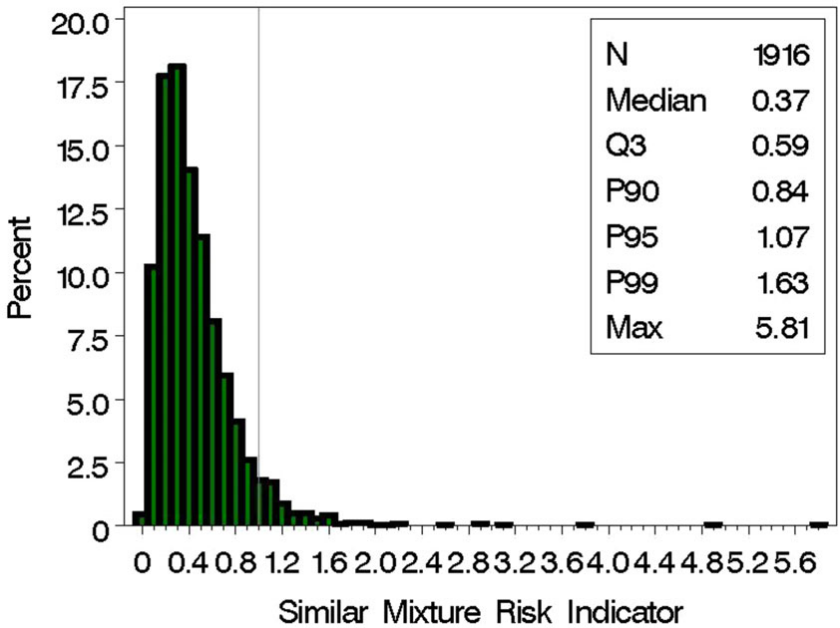
Distribution of similar mixture risk index (SMRI) for the 1,958 pregnant women in SELMA with mixtures sufficiently similar to Mixture S.

For this set of mixtures sufficiently similar (*N* = 1,958), we are interested in determining the relative concentration levels compared to the BMD (from the animal data) of the reference mixture using the SMRI. Here, roughly 15% of the SELMA women have concentrations extreme relative to the BMD identified by the AGD analysis (i.e., identified by SMRI values exceeding 1; Fig. 2), corresponding to about 13% of the total population of 2,313 pregnant women.

**Figure 3:**
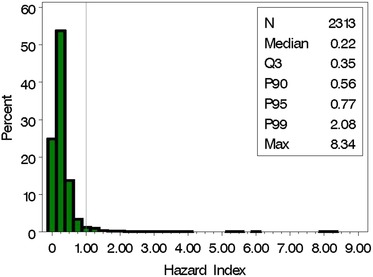
Hazard index for MBP, MBzP, DEHP, and DINP using reference doses from published BE and HBM values as given in Table VI.

In comparison, under the assumption of additivity for the four phthalates and the BE values provided in Table VI, roughly 3% of the 2,313 pregnant women in SELMA have HI values that exceed 1.0, indicating that further evaluation should be conducted (Fig. 3).

**Table VI:** Published Guideline Values (BE and HBM Values) for Urinary DBP, BBzP, DEHP, and DINP (µg/L) Used to Calculate the Hazard Index

		Observed Concentration in SELMA (*N* = 2,313)		
Diester	Metabolite	95th Percentile	99th Percentile	Published BE Values^a^
DBP	MBP	233	520	2,700
BBzP	MBzP	101	237	3,800
DEHP	Sum of 4 metabolites in Table III	191	603^b^	400^c^
DINP	Mono‐carboxyoctyl phthalate (MCOP)	78	245	390

a Aylward et al. (2013).

b 1.6% of the SELMA mothers had higher levels than BE.

c HBM‐value for DEHP (sum of 2 metabolites) is 300 µg/L where 1.3% of the SELMA mothers had higher levels.

## 4 DISCUSSION

The purpose of this article was to propose a novel method for risk assessment of chemical mixtures, illustrated by a case study. Toward that aim, we have integrated four important parts of environmental health research: (1) epidemiological data from a pregnancy cohort with recorded exposures and health effects in children for identification of “bad actors,” (2) biomonitoring data for construction of a “typical” mixture consisting of these “bad actors,” (3) animal data on describing adverse effects from controlled *in vivo* experiments for identification of dose–response relationships and PODs, and (4) biostatistical analyses to assess the similarity of mixtures and, for those determined to be sufficiently similar to the reference mixture, to construct an index related to the POD and human exposure.

Our case study identified four phthalates (DBP, BBzP, DEHP, and DiNP) as the primary “bad actors” for a shorter AGD in boys. A mixture of these, based on what may be considered a “typical” mixture in the SELMA pregnant women (Mixture S), indicated a BMD of roughly 2.1*X*, which was associated with a 5% decline in AGD of exposed male mice. Furthermore, 85% of the SELMA women have mixtures sufficiently similar to this reference mixture. Finally, the whole mixture strategy for risk assessment utilized the concept of sufficient similarity with the construction of an SMRI, which incorporates both the mixture ratio and the comparison of the concentration estimates to the reference “mixture reference values.” Consideration of the SMRI indicated that roughly 13% of the SELMA women (i.e., 15% of the 85% of SELMA women with mixtures determined to be sufficiently similar to the reference mixture) were comparable to the reference mixture and had extreme levels of the four phthalates based on the BMD in the mouse study. In comparison to the whole mixture approach, the combination strategy for cumulative risk assessment utilized the concept of dose addition and the HI. Assuming additivity, roughly 3% of the SELMA pregnant women have concentrations of phthalates with an HI that exceeds 1.0, necessitating further evaluation and concern.

With this novel whole mixture approach, we found that about 13% of the SELMA women were identified as having an exposure that exceeded levels considered safe. In contrast, using the traditional mixture approach assuming additivity, roughly 3% of the women were at risk. Notably, all SELMA women had urinary phthalate levels below existing HBM/BE values for three of the four phthalates, while 1.6% of the women had levels above BE for DEHP (Table VI).

We suggest that this novel approach may be more relevant than single chemical risk assessment since it combines epidemiological and *in vivo* animal studies, and takes into consideration the fact that humans are routinely exposed to mixtures of chemicals, not single compounds one at a time.

Mixture S identified in Step 2 was based on the DI estimated phthalate diesters, following the general strategy developed and used by the EDC‐Mix Risk Consortium (Birgersson et al., 2017). However, in reality, the mixtures constructed for children´s neurodevelopment (Mixture N) and growth (Mixture G), respectively, including 12 phthalate metabolites, alkyl phenols (BPA and triclosan), and eight PFASs, were evaluated in both *in vivo* and *in vitro* assays. The team agreed to use metabolites for the phthalates as a substitution for the diesters for two reasons: (1) the active components are the monoesters in humans and are rapidly metabolized from the diesters so that the appropriate component for the *in vitro* studies was the monoesters; and (2) there was a problem in constructing the mixtures with high concentrations of phthalates (requiring cost‐prohibitive quantities) and low concentrations of the PFASs.

To keep the relative proportions of the phthalates comparable in the three mixtures (S, N, and G), the actual mixture constructed for Mixture S was a mixture of phthalate monoesters and not diesters. We present the analyses here in terms of diesters under the assumption of approximately similar proportions. However, when we calculate the distance estimates using monoesters instead of diesters in comparison to the reference mixture defined as monoesters, there are more women with mixtures sufficiently similar to Mixture S: 94% of the women were determined to be sufficiently similar to Mixture S and roughly 9% of the women in the similarity set have SMRI values that exceed 1, that is, 8% of all the SELMA mothers compared with about 13% in the analyses using monoesters.

AGD is a sexually dimorphic feature both in humans and rodents and is considered an index of prenatal exposure to androgen‐disrupting chemicals. Normally, AGD is approximately double in males than in females, but this ratio is often altered by *in* *utero* acting EDCs, including phthalates (Salazar‐Martinez et al., 2004). The detected reduction of AGD in young male mice offspring prenatally exposed to Mixture S of the present study is thus indicative of the antiandrogenic properties of at least some components of the mixture. Previous studies in male mice offspring perinatally exposed to a single component of Mixture S (DEHP or DBP) have shown analogous reductions in AGD (Barakat et al., 2017; Moody et al., 2013; Stenz, Escoffier, Rahban, Nef, & Paoloni‐Giacobino, 2017) at daily doses ranging from 20 µg to 500 mg/kg BW. In the present study, mice mothers treated with the 10*X* dose were daily exposed to 2.6 mg/kg BW of the Mixture S. However, direct comparisons cannot be made due to the inclusion of monoesters in Mixture S and to potential differences in the impact of chemicals acting as single components or within a mixture, where synergistic or antagonistic relationships can develop.

An overall strength with this whole mixture approach is that we have integrated human‐based epidemiology with experimental‐based toxicology in a mouse model. Such an approach will bring human relevance into the study. We have exposed mice dams at doses relevant to the exposure detected in a Swedish population of pregnant women (SELMA). Chemical analysis of the mice serum during pregnancy verified that the steady‐state levels of phthalates achieved in their blood were actually similar to those estimated in SELMA women (see the appendix in Supporting Information). We further found in the animal study that doses between 1*X* and 10*X* were associated with about a 5% decline in AGD. Interestingly, in a single‐compound analysis in the SELMA study, a similar decline in AGD was observed among 194 boys at 21 months of age (about 5%) when the prenatal DiNP exposure was increased roughly 10 times (Bornehag et al., 2015). A further strength of the study is the sample size for sufficient similarity and SMRI calculations where we could use the full cohort of SELMA, i.e., 2,313 pregnant women. Finally, the chosen design enabled us to compare results from three different approaches: the sufficient similarity/SMRI, a more traditional additive model, and, finally, a compound‐by‐compound approach.

However, the study also has limitations. The sample size of children with AGD data was small (*N* = 184) with related power problems for the statistical modeling. Another limitation is that the human urinary samples were collected more than seven years ago—with trends in urinary phthalate levels changing over short periods of time, as shown in a recent study in SELMA (Shu et al., 2018). Although this is not an evaluation of the current exposure, the strategy is demonstrated here.

### 4.1 CONCLUSIONS

This is the first case study to show the proof of concept for a systematic integration of epidemiological and experimental evidence with mixture risk assessment strategies, enabled by the EDC‐MixRisk Consortium. Using such a whole mixture approach, we could find a higher rate of pregnant women under risk (13%) when comparing with the data from more traditional models of additivity (3%), and a compound‐by‐compound strategy (1.6%), which is the most used risk assessment procedure.

## Supporting information


**APPENDIX**: Calculation of Levels of Phthalate Metabolites Levels in Mice Exposed to Mixture S
