# Supplementary material for: A Novel Approach to Chemical Mixture Risk Assessment—Linking Data from Population‐Based Epidemiology and Experimental Animal Tests
Source: Risk Anal. 2019 Jun 7;39(10):2259–71. doi: 10.1111/risa.13323 (PMC6973107; doi:10.1111/risa.13323)
Supplement: Supplementary file 1 — APPENDIX: Calculation of Levels of Phthalate Metabolites Levels in Mice Exposed to Mixture S [file RISA-39-2259-s001.docx]

**APPENDIX Calculation of levels of phthalate metabolites levels in mice exposed to Mixture S**

1. We used the mean mice serum levels of each phthalate (MBP, MBzP, MEHP, MiNP), as measured in pregnant mice serum

| Metabolite | MW | 100X average serum levels  (ng/ml) | 10X average serum levels  (ng/ml) |
| --- | --- | --- | --- |
| MBP | 222 | 302 | 45 |
| MBzP | 256 | 251 | 24 |
| MEHP | 278 | 386 | 33 |
| MiNP | 290 | 266 | 22 |

1. We transformed these mean values into moles/L by dividing each with the respective molecular weight (MW).
2. We then extrapolated these 100X and 10X values to 1X (divided molarities by 100 and 10 respectively). This led to the following table:

| Chemical | Estimated  geometric mean in SELMA (1X)  (mol/L) | Serum levels in mice  (mol/L) | |
| --- | --- | --- | --- |
|  |  | Calculated from 100X | Calculated from 10X |
| MBP | 2.3 E-08 | 1.36 E-08 | 2.00 E-08 |
| MBzP | 1.1 E-08 | 0.98 E-08 | 0.94 E-08 |
| MEHP | 1.5 E-08 | 1.40 E-08 | 1.18 E-08 |
| MiNP | 2.1 E-08 | 0.92 E-08 | 0.76 E-08 |
| Total | 7.0 E-08 | 4.66 E-08 | 4.88 E-08 |

1. As can be seen from the above table, the calculated levels in mice, based on values from 100X or 10X animals are quite close (4.66 and 4.88 E-08). Their mean is 4.77 E-08 mol/L.
2. This means that when we fed the 10X dose of Mixture S we ended up with 6.8 (~7)X SELMA levels and when we fed the 100X Mixture S we ended up with 68(~70)X SELMA levels.
3. We can assume that if we had fed mice with X1 mixture, we would have in mice serum 0.68X SELMA levels. Thus, the assumptions for calculating serum calculations in SELMA are approximately similar to those observed in mice.
